# Administration of an extrasynaptic δ-subunit GABA_A_ receptor agonist in the prelimbic cortex attenuates methamphetamine sensitisation and relapse to methamphetamine-seeking behaviour in male rats

**DOI:** 10.1177/02698811261430509

**Published:** 2026-03-28

**Authors:** Maarten van den Buuse, Jessica Roberts, Michael T. C. Hunter, Gracie L. Hay, Nicholas A. Everett, Sarah J. Baracz, Travis A. Wearne, Jennifer L. Cornish

**Affiliations:** 1 School of Psychology and Public Health, La Trobe University, Melbourne, VIC, Australia; 2 Behavioural Neuropharmacology Laboratory, Faculty of Medicine, Health and Human Sciences, School of Psychological Sciences, Macquarie University, Sydney, NSW, Australia; 3 School of Psychology, University of Sydney, NSW, Australia

**Keywords:** methamphetamine, psychosis, addiction, relapse, GABA, prelimbic cortex

## Abstract

**Aims:**

Chronic methamphetamine (METH) intake is associated with a high risk of psychosis and high susceptibility to relapse following abstinence. Prelimbic cortex (PLC) extrasynaptic GABA_A_ receptors containing the δ-subunit may be involved in the long-term effects of METH. We therefore investigated the effects of intra-PLC injection of the δ-subunit GABA_A_ receptor agonist 4,5,6,7-tetrahydroisoxazolo (5,4-c)pyridine-3(-ol) (THIP), on METH sensitisation, a model of METH psychosis, or relapse to METH seeking behaviour following extinction of intravenous self-administration.

**Methods and results:**

In Experiment 1, male rats received 1 week of chronic treatment with METH (1 mg/kg days 1 and 7, 5 mg/kg days 2–6) or saline, followed by 14 days withdrawal. THIP (0, 0.1, 0.3 or 1.0 μg per hemisphere) was then micro-injected into the PLC prior to challenge with METH (1 mg/kg) or saline. Acute METH challenge enhanced locomotor hyperactivity in METH-pretreated rats, confirming sensitisation. THIP administration into the PLC dose-dependently reduced the effect of acute METH challenge in METH-pretreated rats. In Experiment 2, rats were trained to intravenously self-administer METH, followed by 2 weeks of extinction. Rats were then given a METH priming injection or exposed to cues associated with METH self-administration, both of which reinstated responding on the lever previously paired with METH delivery. At the 0.3 µg dose, injection of THIP into the PLC reduced reinstatement responding in both paradigms.

**Conclusion:**

Selective activation of extrasynaptic PLC δ-subunit GABA_A_ receptors reduced METH sensitisation and drug primed or cue-induced METH-seeking behaviour. These findings may aid development of effective pharmacotherapies for treating chronic METH use.

## Introduction

Methamphetamine (METH) is a highly addictive psychostimulant that exerts widespread effects on the brain and body (Koob, 1992; Meredith et al., 2005). The rewarding effects of METH have led to high rates of consumption throughout the world (Degenhardt et al., 2017; Jones et al., 2020). While acutely rewarding, long-term METH use produces a range of persistent neurobiological alterations that give rise to detrimental cognitive and psychological deficits (Scott et al., 2007) including a high risk of psychosis-like episodes that resemble schizophrenia (Arunogiri et al., 2018; Hogarth et al., 2022; Wearne and Cornish, 2018) and adverse physical health concerns including cardiovascular problems, stroke, dental decay, malnourishment and respiratory problems (Darke et al., 2008). Chronic use often progresses to a compulsive pattern of use characterised by recurrent relapse and an inability to abstain from repetitive use (Cruickshank and Dyer, 2009).

Following chronic METH use, several neurobiological alterations occur that give rise to enduring cognitive and psychological changes. Long-term use causes neurotoxicity to monoamine systems, resulting in a persistent widespread reduction of monoamine neurotransmission, especially dopamine (Cruickshank and Dyer, 2009; Hogarth et al., 2022; McCann et al., 2008; Meredith et al., 2005). Along with altered monoamine function, structural abnormalities and reductions in grey and white matter densities in several limbic, subcortical and prefrontal areas have been measured (Nakama et al., 2011; Thompson et al., 2004). These neurobiological changes contribute to cognitive and affective impairments and increase the risk of METH dependence and METH-induced psychoses. Treatments yield positive short-term outcomes, but their long-term effectiveness remains limited prompting the need for efficacious pharmacological treatments that target underlying markers of addiction and relapse to METH dependence as well as METH psychosis (Arunogiri et al., 2018; Brackins et al., 2011; Van den Oever et al., 2010).

The prefrontal cortex (PFC) is involved in the development of METH psychosis and is central to the transition from impulsive drug taking to compulsive, uncontrollable use (Goldstein and Volkow, 2011; Hogarth et al., 2022; Limpens et al., 2015). Long-term exposure to METH is associated with degraded frontal cortical cognitive functioning such as deficits in executive functioning, processing speed, memory and attention (Scott et al., 2007). Following chronic METH exposure, similar frontal impairments are observed in the rodent medial PFC (mPFC); a region analogous to the human anterior cingulate cortex (Heidbreder and Groenewegen, 2003; Milad et al., 2007). The rodent mPFC is located along the medial wall of the PFC (Heidbreder and Groenewegen, 2003), and plays a crucial role in modulating a variety of rat behaviours, in particular relapse to METH-seeking (Hiranita et al., 2006; Rocha and Kalivas, 2010; Volkow et al., 2012). The rat mPFC can be divided into four subregions; the anterior cingulate cortex, prelimbic cortex (PLC), infralimbic cortex (ILC) and medial agranular cortex (Heidbreder and Groenewegen, 2003; Hoover and Vertes, 2007). The PLC and ILC are thought to evoke opposing roles in drug-seeking behaviour, whereby the PLC promotes and drives drug-seeking during self-administration and relapse, whilst the ILC inhibits drug seeking during withdrawal and abstinence (Limpens et al., 2015; Willcocks and McNally, 2013), although recent research suggests more nuanced involvement in these behaviours (Moorman and Aston-Jones, 2023). Given the dominant drive of the PLC to promote addictive drug behaviours, it remains a candidate region for research into drug addiction and relapse vulnerability.

In the rodent and human brain, the PLC has widespread reciprocal connections to sub-cortical regions in the mesocorticolimbic dopamine pathway such as the ventral tegmental area, nucleus accumbens (NAc), amygdala and striatum (Hoover and Vertes, 2007). This neural substrate regulates an array of behaviours and cognitive processes, in particular response inhibition, which is known to be severely impaired following chronic METH use (Hoover and Vertes, 2007). For example, pharmacological inactivation or lesioning of the PLC impaired impulse control in rats (Chudasama et al., 2003) and slowed response inhibition on the stop-signal task (Bari et al., 2011). In addition to an involvement of inhibitory control, PLC dysfunction impaired goal-directed behaviour and induced rigid stimulus-response habits (Killcross and Coutureau, 2003). These studies implicate the PLC in executing inhibitory cognitive control and goal-directed evaluations over behaviour, and dysfunction of the PLC induced by prolonged substance abuse may result in uncontrollable drug taking that remains insensitive to adverse consequences (Limpens et al., 2015; Perry et al., 2011; Pelloux et al., 2013).

γ-aminobutyric acid (GABA) is a major inhibitory neurotransmitter, well-described for its involvement in modulating synaptic signalling, where deficiencies in PFC GABA signalling have been associated with METH addiction (Wearne and Cornish, 2019). For example, following chronic METH exposure, prefrontal GABA concentrations are rapidly reduced (Bu et al., 2013) and glutamate dehydrogenase (GAD67), the primary enzyme involved in the synthesis of GABA, is down-regulated in the PFC (Wearne et al., 2015; Zhang et al., 2006). PLC inhibition blocked METH-primed and METH-cued reinstatement to METH-seeking behaviour in rats (Rocha and Kalivas, 2010) and non-specific activation of GABARs, to inhibit the PLC, eliminated METH-primed and cue reinstatement to METH-seeking behaviours (Rocha and Kalivas, 2010).

GABA_A_Rs containing the δ subunit display unique functional properties. These GABA_A_Rs, are comprised of two α and β subunits, and one δ subunit and pertain exclusively outside the synapse (extrasynaptic) on somatic, dendritic and axonal regions of neuronal membranes (Brickley and Mody, 2012; Meera et al., 2011). δ-subunit GABA_A_Rs are less temporally restricted and their primary action is to regulate tonic inhibition (Farrant and Nusser, 2005), increasing the excitatory threshold needed for a cell to generate an action potential (Farrant and Nusser, 2005). Given this, extrasynaptic GABA_A_Rs can profoundly affect neural excitability within the CNS (Whissell et al., 2015). Therefore, enhancing δ-GABA_A_R-mediated tonic inhibition within PLC could counteract the disinhibition produced by chronic METH exposure and reduce relapse-related behaviours.

At GABA_A_Rs, GABA is a potent full agonist, however at extrasyntaptic δ-subunit GABA_A_Rs it is only a partial agonist when compared to the ‘superagonist’ behaviour of Gaboxal (4,5,6,7-tetrahydroisoxazolo (5,4-c)pyridine-3(-ol), THIP; Meera et al., 2011). THIP is a selective ligand for extrasyntaptic GABA_A_Rs, due to its highly effective activation and greater binding affinity at GABA_A_Rs containing the δ-subunit compared to GABA itself (Meera et al., 2011; Mortensen et al., 2010). These properties make THIP an ideal drug to investigate the effects of tonic GABA activity within the PLC and its subsequent influence over relapse propensity. Maguire et al. (2014) revealed that injections of THIP administered exogenously, or locally into the NAc, reduced the preference for rats to enter an environment previously associated with cocaine or amphetamine (AMPH) use. In contrast, global depletion of the receptor in δ-subunit knock-out mice was not effective in reducing METH reward preference (Siivonen et al., 2018), yet appeared to show enhanced sensitized activity compared to wild type mice following repeated METH injections. This evidence suggests that restoring tonic GABA current, via pharmacological activation of δ-subunit GABA_A_Rs, may reduce METH-related behavioural effects, and although the δ-subunit GABA_A_Rs may not be required to express the rewarding effects of METH, they may be important modulators to dampen METH sensitisation.

The aim of the present study was to investigate the hypothesis that chronic METH-induced behaviours may be reduced by the activation of δ-subunit GABA_A_Rs in the PLC. We used two common animal models of the long-term effects of METH: METH-induced locomotor sensitisation (Experiment 1) as a model of sensitivity to METH-induced psychosis (Featherstone et al., 2007; Hogarth et al., 2022; Robinson and Berridge, 2001; Stewart and Badiani, 1993; Wearne and Cornish, 2018) and METH- and cue-induced reinstatement following extinction of long-term METH intravenous self-administration (IVSA; Experiment 2) as a model of relapse of METH taking (Epstein et al., 2006; Hyman and Malenka, 2001; Steketee and Kalivas, 2011).

## Methods

### Animals

Adult male (approx. 3 months old) Sprague Dawley rats (initially *n* = 64 for Experiment 1; *n* = 32 for Experiment 2), weighing between 250 and 300 g on arrival, were sourced from the Animal Research Centre in Perth, WA. The rats were housed together in groups of four with food and water available ad libitum. Home cages (cage size: 62.9 cm × 40 cm × 25.8 cm) consisted of a white plastic tub with a mesh wire roof lined with recycled paper bedding. The rat housing facility was lit on a 12-hour light/dark cycle (lights on at 6:00 hours), with the room temperature retained at 21 ± 1°C. All experiments were conducted during the light period. After a 7-day period of acclimation, the rats were handled daily for 7 days to familiarise them to the experimenter. All procedures were conducted in compliance the Australian Code of Practice for the Care and Use of Animals for Scientific Purposes (National Health and Medical Research Council of Australia, 2013), and approved by the Macquarie University Animals Ethics Committee.

### Drugs

We obtained 4,5,6,7-Tetrahyrdoisoxazolo[5,4-*c*]pyridine-3-ol hydrochloride (Gaboxadol hydrochloride, or THIP) from Sigma-Aldrich (Castle Hill, NSW, Australia). METH hydrochloride was purchased from the Australian Government Analytical Laboratories (Pymble, NSW, Australia), while physiological saline (0.9%) was acquired from Baxter Healthcare (Old Toongabbie, NSW, Australia). METH and THIP were both dissolved in 0.9% saline. For intraperitoneal (i.p.) injection, METH was delivered at a volume of 1 mL/kg. For IVSA in Experiment 2, METH solutions were filtered through a Millipore syringe filter (0.22 µm) prior to use in the intravenous lines. Each dose of THIP was delivered at a volume of 0.5 µL/brain hemisphere and administered via 11 mm intracranial (i.c.) microinjectors made of 33G steel hypodermic tubing (VitaNeedle, Needham, USA), inserted into bilateral indwelling 26 G cannulae (10 mm) into the PLC.

### Experimental procedure

**Figure 1.**
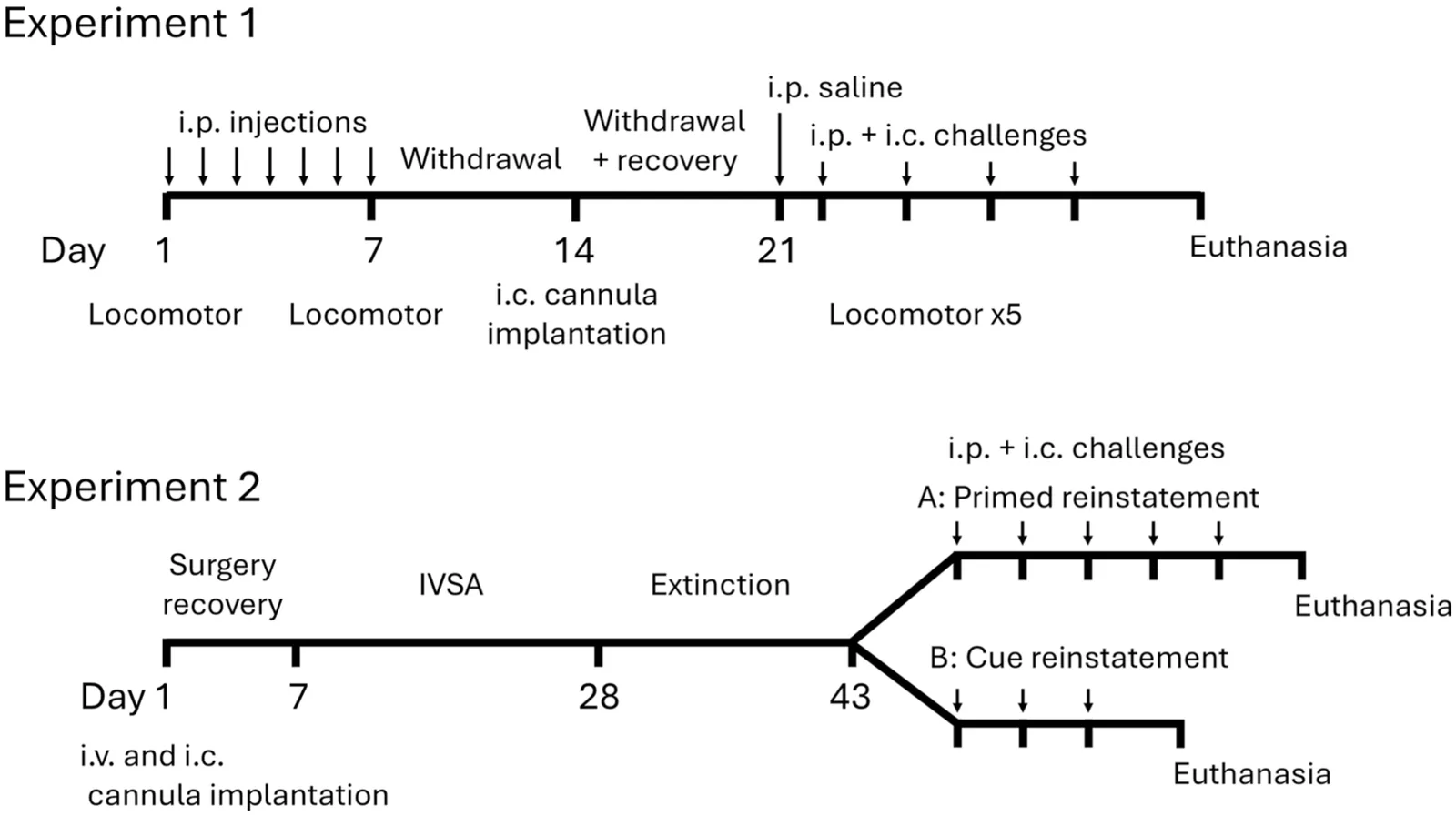
Timeline of the experiments. IVSA = intravenous self-administration. Arrows indicate i.p. or intracerebral (i.c.) injections. Note that experiment 2 consisted of 2 groups—METH-primed reinstatement (Experiment 2A) or cue-induced reinstatement (Experiment 2B).

Figure 1 shows the timeline of the procedure for locomotor sensitisation and microinjection challenge days in Experiment 1 and the IVSA procedure and reinstatement test days for Experiment 2.

In Experiment 1, following chronic METH administration and withdrawal (Figure 1), the rats underwent surgery to implant bilateral i.c. cannulae (26 G; 10 mm) to administer THIP into the PLC. In Experiment 2, prior to the IVSA procedure (Figure 1), the rats underwent surgery to implant a chronic indwelling catheter into the right jugular vein as previously described (Baracz et al., 2015; Motbey et al., 2013), followed by bilateral cannula implantation into the PLC (Baracz et al., 2015). The rats were anaesthetised with 3% isoflurane in oxygen and given a subcutaneous (s.c.) injection of the anti-inflammatory analgesic, Carprofen (5 mg/kg), and saline (1 mL s.c. each flank) to ensure hydration.

To enable i.c. cannula implantation, anesthetized rats were then placed in a stereotactic frame (Baracz et al., 2015). Cannulae were bilaterally placed 1mm above the PLC (anterior posterior AP, +3.2 mm, medial lateral ML, +1.0 mm, dorsoventral DV, −2.6 mm; relative to Bregma; Paxinos and Watson, 2013) through small holes drilled in the skull. Four jeweller’s screws inserted into the skull and acrylic cement secured the cannulae in place. Stainless steel stylets (obturators) measuring 10 mm in length were inserted into the cannulas to prevent occlusion. After recovery, rats were returned to the housing facility and remained in individual housing for 2 days, before being transferred back into group housing chambers.

Post-operative recovery consisted of analgesic administration of s.c. Carprofen injections on the first 2 days post-surgery, and flushing of jugular catheters with a heparin (60 IU in 0.2 mL) and cephazolin sodium (20 mg in 0.2 mL) solution, to prevent infection and ensure catheter patency. Procedures in Experiment 1 were similar, except for intravenous catheter implants.

Injections into the PLC were 0.5 μL volume per hemisphere. Microinjectors were constructed using 33 G steel hypotubing, and were connected to plastic tubing attached to 1 μL Hamilton syringes (Sigma-Aldrich, Castle Hill, NSW, Australia). Microinjectors extended 1 mm beyond the length of the cannulae and the rate of microinjection was controlled for by an infusion pump (KD Scientific, Holliston, MA, USA) at a rate of 0.5 μL/min. Injectors were left in the cannulae for an additional 30 seconds to allow diffusion of the injected solution, after which 70% alcohol-cleaned obturators were re-inserted.

### Experiment 1: METH sensitisation, withdrawal and challenge

To measure locomotor activity, we used four Infrared Actimeter chambers (Imetronic, Pessac, France), each containing 8 individual, clear plastic activity cages (36 cm (L) × 24 cm (W) × 19 cm (H)). These cages contained wire mesh flooring and roofing, with infrared sensors located on the sides of the cages to record locomotor activity, expressed as number of beam breaks. Prior to all substance administrations, rats were given 15 minutes within their allocated cage to habituate, before i.c and i.p injections and their activity recorded for the following 60 minutes.

METH pretreatment was carried out as previously described (Jaehne et al., 2023; Wearne et al., 2015). METH doses were given at 1 mg/kg on locomotor testing days (days 1 and 7), and 5 mg/kg on non-testing days (days 2–6). Control rats received saline injected at a volume of 1 mL/kg, i.p. Following the chronic treatment, rats were housed in their homecages and withdrawn from METH/Saline for 2 weeks (Figure 1). On day 14, rats underwent i.c. surgery and were given 7 days to recover post surgery before challenge administration of METH or saline. On day 21, following withdrawal and recovery from surgery, rats were again placed in the locomotor chambers and after 15 minutes of habituation were given an i.p injection of saline (1 mL/kg). Baseline locomotor activity was then recorded for 1 hour. At least 24 hours later, rats were given an i.p challenge dose of either METH or saline, generating four possible conditions depending on prior treatment (Saline/Saline, Saline/METH, METH/Saline, METH/METH, *n* = 16). This was done on four challenge days with i.p. saline or METH (1 mg/kg) administered directly following a microinjection into the PLC of vehicle (Saline) or one of three doses of THIP (0.1, 0.3 or 1.0 μg per hemisphere, or 0.2, 0.6, 2 μg total), according to a Latin-square design to ensure all rats received all THIP doses. Following each challenge treatment, locomotor activity was measured for 60 minutes. A minimum of 48-hours rest in home cages separated each microinjection challenge test to allow for recovery and complete washout of drugs.

### Experiment 2: IVSA, extinction and reinstatement

For animals in both METH-primed (Experiment 2a) or cue-induced (Experiment 2b) reinstatement groups, IVSA was conducted as described previously (Baracz et al., 2015). Briefly, after recovery from surgery, rats were trained to lever press for METH in daily 2-hour sessions. Operant chambers (MedAssociate, VT, USA) contained two retractable levers (1 active for drug delivery and 1 inactive), a white stimulus light above each lever, a dimly lit house light on the opposing wall and infrared photo beam detectors to measure locomotor activity. Where cue-induced reinstatement was assessed (Experiment 2b), rats were also exposed to a peppermint oil odour that acted as an environmental cue for METH availability. A syringe located outside the chamber was fitted to each infusion pump and attached to polyethylene tubing (PE 50) encased in a steel spring tether that connected to the rat’s intravenous catheter. MED-PC IV software recorded active and inactive lever presses, number of infusions and locomotor activity (MedAssociate, VT, USA).

During each 2-hour session rats were trained on a fixed ratio one (FR1) schedule of reinforcement, under which active lever presses resulted in a 3-second intravenous infusion of METH (0.1 mg/kg/infusion; 0.05 mL). Following each infusion, a 20 second time-out period initiated where the house light turned off and the light above the active lever became illuminated, and further lever presses were without consequence. This reduced the risk of overdose, allowed the effects from the drug infusion to be experienced before re-administering and enabled the time out stimuli (lever light and house light) to symbolise drug availability. The allocation of the active and inactive levers was counterbalanced across chambers. The session concluded after a 2-hour period or a maximum number of 60 infusions had been reached and was indicated by lever retraction and termination of the house light. Following session completion, rats were disconnected from the infusion line, catheters flushed with 0.2 mL of cephazolin sodium (100 mg/mL) dissolved in a heparinised saline solution (300 IU/mL) and returned to group housing. Rats undertook METH self-administration training for a total of 15 sessions, conducted 5 days a week.

Extinction consisted of 15 consecutive days of 2 hours sessions where METH administration was no longer available. For METH-primed reinstatement (Experiment 2a), presses on the active lever resulted in no contingent METH infusion, but still initiated a 20 second time-out period where the house light turned off and the light above the active lever became illuminated. For cue-reinstatement (Experiment 2b), rats were similarly not exposed to METH infusion following an active lever press. However, cues that were associated with METH availability; such as the peppermint oil, illumination of active lever light and termination of house light during active lever responding, were also not available during the extinction sessions. Extinction criteria were met when no significant preference for the active over the inactive lever was present and/or there were less than 10 active and inactive lever presses made per session over the last two consecutive days of extinction.

Prior to beginning each reinstatement session, rats received an i.c. bilateral infusion of either THIP or vehicle, as described for Experiment 1. Each reinstatement test session was separated by at least a 48-hour washout period with daily extinction conditions to ensure THIP had completely metabolised (Larsen et al., 2010) and METH prime/cue-induced lever pressing had re-extinguished prior to the next test.

To assess METH-primed reinstatement (*n* = 16), a total of five sessions was conducted. A within-subjects design was used, meaning all rats underwent all treatment conditions. On each of the first four METH-primed reinstatement periods, rats initially received an intracranial microinjection of either vehicle (0.9% saline) or one of three THIP doses (0.1, 0.3, 1.0 μg THIP per brain hemisphere, or 0.2, 0.6, 2 μg total) in 0.5 μL of saline. Microinjections of vehicle and THIP dosages were counterbalanced using a Latin square design to preclude any ordering effects of drug treatment. Immediately following microinjection, all rats received an i.p. injection of METH (1 mg/kg in a 1 mL/kg volume) and were placed in their respective operant chambers, where reinstatement behaviour was monitored for a 2-hour period. Reinstatement conditions were identical to IVSA sessions, apart from unavailability of METH on active lever depression. On the fifth reinstatement day, the rats received only an i.p. injection of METH to ensure that a METH prime still reinstated METH-seeking behaviour after repeated microinjections into the PLC.

To assess cue-induced reinstatement (*n* = 16), a total of three reinstatement sessions were conducted. A within-subjects design was utilized, whereby all rats received all treatment conditions. On the first two cue-reinstatement periods, rats received an i.c. microinjection of either vehicle or THIP at the dose of 0.3 μg THIP/per brain hemisphere (or 0.6 μg in total), based on the effective dose in Experiment 2a. Vehicle and THIP were counterbalanced across the 2 days to preclude any ordering effects of drug treatment. Immediately following i.c. treatment, the rats were placed into their respective operant chambers and re-exposed to the cues associated with METH availability in the IVSA period. These cues were peppermint oil smell, illumination of active lever light and termination of house light upon active lever depression. On the third and last reinstatement session, rats received a bilateral infusion of THIP without presentation of METH-associated cues to determine that THIP alone did not alter or reinstate lever press activity.

### Euthanasia and histology

Once testing was completed, rats were anaesthetised with a high dose of pentobarbital sodium (135 mg/mL i.p.), then perfused transcardially with 0.9% saline (40 mL) followed by 10% formalin (40 mL). The brains were removed and fixed in a formalin solution for a minimum of 7 days before histology was performed. Sixty μm coronal brain sections were taken across the microinjection site to verify correct bilateral cannula placements (Paxinos and Watson, 2013). Only data from rats with verified bilateral cannula placements in the PLC were included in the statistical analysis (Supplemental Figure).

### Data analysis

SPSS version 21 (IBM, Chicago, IL) was utilized to conduct the data analysis, with statistical significance set at *p* < 0.05. All assumptions were assessed and met. Upon violations to sphericity, as indicated by Mauchley’s test, the Greenhouse–Geisser correction was applied where relevant.

### METH sensitisation

To test sensitisation over the initial 7-day administration period, two-way analysis of variance (ANOVA) was used with day and treatment as factors to compare locomotor activity of METH-treated and saline-treated rats on days 1 and 7.

To compare the effects of THIP microinjections on challenge administration days, one-way repeated measures ANOVA was used to analyse the effect of local PLC microinjections of vehicle or THIP on locomotor activity. Hypotheses were set a priori and planned pairwise comparisons were conducted, comparing PLC microinjections of THIP doses set at 0.1 µg/per hemisphere, 0.3 µg/per hemisphere and 0.1 µg/per hemisphere to vehicle.

### IVSA and extinction

Repeated-measures ANOVA was also used to ensure that all rats had learned to acquire METH IVSA. The average number of active lever presses and number of daily infusions on the first three and last three self-administration sessions were directly compared. The number of active and inactive lever presses on the final self-administration day, was also compared to ensure rats had learned to differentiate between the two levers. Rats that failed to acquire METH self-administration were excluded from further analysis. Similar comparisons were used to assess if the rats had extinguished their METH-seeking behaviour before reinstatement testing commenced. Specifically, the average number of active lever presses on the first three and last three extinction days were directly compared. Additionally, to ensure that rats had extinguished METH IVSA by displaying no preference for the active over the inactive lever, the mean number of active versus inactive lever presses on the last 3 days of extinction was directly compared.

### Reinstatement testing

Repeated-measures ANOVA was used to compare active responding for the vehicle reinstatement day and extinction day prior, as well as active and inactive responding on the vehicle reinstatement day. If rats failed to reinstate to active lever responding under vehicle-pretreated METH reinstatement they were excluded from the final analysis. Repeated-measures ANOVA was used to analyse the effect of local PLC microinjections of vehicle or THIP on active lever presses and locomotor activity to an acute METH prime injection. Hypotheses were set a priori and planned pairwise comparisons were conducted, comparing PLC microinjections of THIP doses set at 0.1 μg/per hemisphere, 0.3 μg/per hemisphere and 0.1 μg/per hemisphere to vehicle, on either variables of active lever press or locomotion. An additional paired *t*-test was utilized to compare active lever responding on the last (fifth) METH-primed reinstatement day to the extinction day prior. This certified that repeated microinjections did not confound the ability of a METH priming injection to induce reinstatement of drug-seeking behaviour.

Repeated-measures ANOVA was also used to compare the effect of PLC vehicle and THIP (0.3 µg/hemisphere) microinjections on the number of active lever presses in response to METH-associated cues. To verify that THIP alone did not reinstate any METH-seeking behaviours, the number of active lever pressing between the last (third) THIP-alone reinstatement day and the extinction day prior was similarly compared.

## Results

### Experiment 1: METH sensitisation

Locations of the bilateral cannulas implanted into the PLC are presented in Supplemental Figure 1. Incorrect placement of cannulas outside the PLC meant that three animals were excluded from analysis. Additionally five rats were excluded due to obstructed cannulas. Final numbers for each group of analysis resulted in METH-METH (*n* = 12), saline-METH (*n* = 16), METH-saline (*n* = 12), and saline-saline (*n* = 15).

### Induction of METH sensitisation

**Figure 2.**
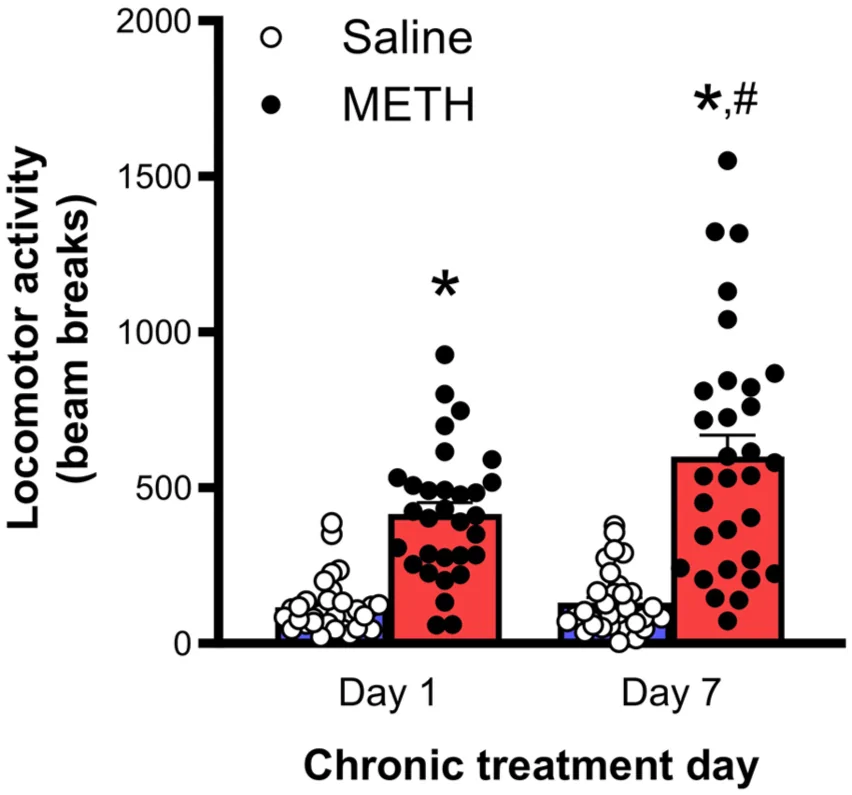
Locomotor hyperactivity sensitisation following 1 week of chronic treatment with METH. Acute METH increased locomotor activity (red bars) and this effect was enhanced following 7 days of treatment. *p < 0.05 for difference with saline; ^#^*p* < 0.05 for difference with day 1. Data are mean ± SEM.

The rats were pretreated with either saline or METH. Analysis of locomotor activity measured on days 1 and 7 of the chronic treatment period showed a main effect of both treatment day (*F*(1, 61) = 14.45, *p* < 0.001, η_*p*_^2^ = 0.192) and METH treatment (*F*(1, 61) = 58.4, *p* < 0.001, η_*p*_^2^ = 0.489), as well as a day × treatment interaction (*F*(1, 61) = 10.4, *p* = 0.002, η_*p*_^2^ = 0.146). Further analysis split by treatment day showed that 1 mg/kg METH significantly increased locomotor activity on both days 1 and 7 of the treatment period when compared to saline treatment (*F*(1, 61) = 57.1, *p* < 0.001, η_*p*_^2^ = 0.484 and *F*(1, 61) = 45.2, *p* < 0.001, η_*p*_^2^ = 0.426, respectively). Confirming METH sensitisation, comparison of days 1 and 7 for each treatment group showed that the response to the 1 mg/kg dose was significantly increased by chronic METH treatment (*F*(1, 30) = 12.8, *p* = 0.001, η_*p*_^2^ = 0.299) but there was no significant difference between days 1 and 7 locomotor activity in rats that received chronic saline (Figure 2).

### Effect of intracerebral THIP on METH challenge-induced locomotor hyperactivity

**Figure 3.**
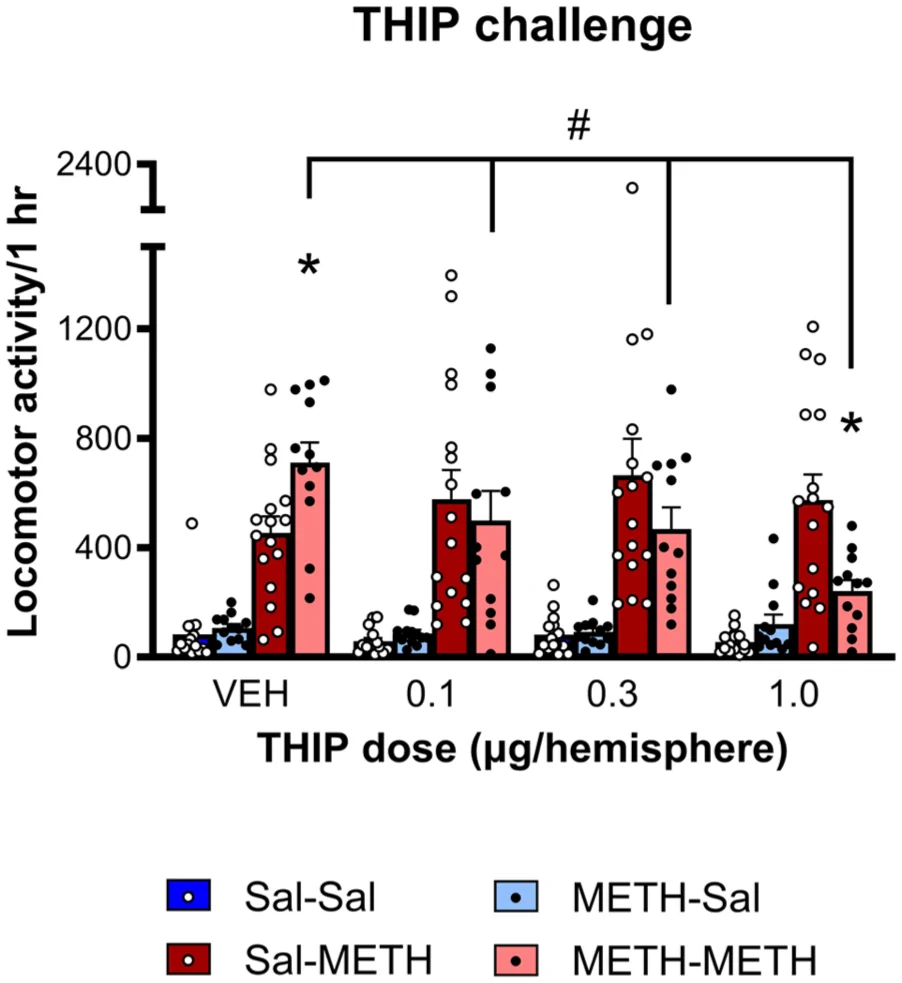
Microinjection of THIP into the PLC reduces acute i.p. METH-induced locomotor hyperactivity following METH pretreatment. ^#^*p* < 0.05 for difference between i.c. THIP treatment and i.c. saline in the METH/METH group; THIP significantly reduced locomotor activity in the METH-METH condition at all doses. *p < 0.05 for difference of acute METH-induced locomotor activity between METH/METH rats and Sal/METH rats. Data are mean ± SEM of *n* = 15 in the saline/saline group, *n* = 12 in the METH/saline group, *n* = 16 in the saline/METH group and *n* = 12 in the METH/METH group.

Locomotor activity in the four pretreatment/treatment groups, Sal/Sal, METH/Sal, Sal/METH and METH/METH, was measured four times, following a Latin-square design of intracerebral injection of vehicle or one of three doses of THIP (Figure 3). Comparing all groups and all THIP doses revealed the expected main effect of acute METH challenge compared to acute saline (*F*(1, 51) = 71.8, *p* < 0.001, η_*p*_^2^ = 0.585) but also an interaction between acute METH, chronic METH pretreatment and THIP dose (*F*(2.5, 126.4) = 5.76, *p* = 0.002, η_*p*_^2^ = 0.102). Further analysis of the data split by treatment group showed no effect of THIP dose in Sal/Sal rats, METH/Sal rats and Sal/METH rats. However, in METH/METH rats there was a main effect of THIP dose (*F*(3, 33) = 8.64, *p* < 0.001, η_*p*_^2^ = 0.440) and planned comparison showed that locomotor hyperactivity following acute METH challenge in the chronic METH group was significantly reduced by intracerebral injection of all doses of THIP (0.1 µg/hemisphere: *F*(1, 11) = 4.92, *p* = 0.048, η_*p*_^2^ = 0.309; 0.3 µg/hemisphere: *F*(1, 11) = 7.44, *p* = 0.020, η_*p*_^2^ = 0.403; 1.0 µg/hemisphere: *F*(1, 11) = 39.2, *p* < 0.001, η_*p*_^2^ = 0.781). The data were also split by THIP dose and analyzed by univariate ANOVA followed with Bonferroni-corrected multiple comparisons. Following i.c. vehicle injection, acute METH challenge increased locomotor activity compared to acute saline, and this effect was significantly greater in rats pretreated with METH (Figure 3). There were no differences in the effect of acute METH following i.c. injection of either 0.1 or 0.3 µg/hemisphere THIP, i.e. METH-induced locomotor hyperactivity was no longer sensitized. In contrast, in rats i.c. injected with 1.0 µg/hemisphere THIP and pretreated with METH, the effect of acute METH challenge was significantly reduced (Figure 3).

### Experiment 2: METH reinstatement

Locations of the bilateral cannulas implanted into the PLC are presented in Supplemental Figure 1. Of the original 32 rats, for the final analysis 10 were included for the METH primed reinstatement component of this experiment and 9 were used for cue-induced reinstatement. Rats were omitted based on compromised catheter patency (2), an inability to acquire METH self-administration (1), failure to extinguish (1) or reinstate to METH-seeking behaviour (2) and misplaced (6) or blocked (1) intracranial microinjections.

### IVSA and extinction

**Figure 4.**
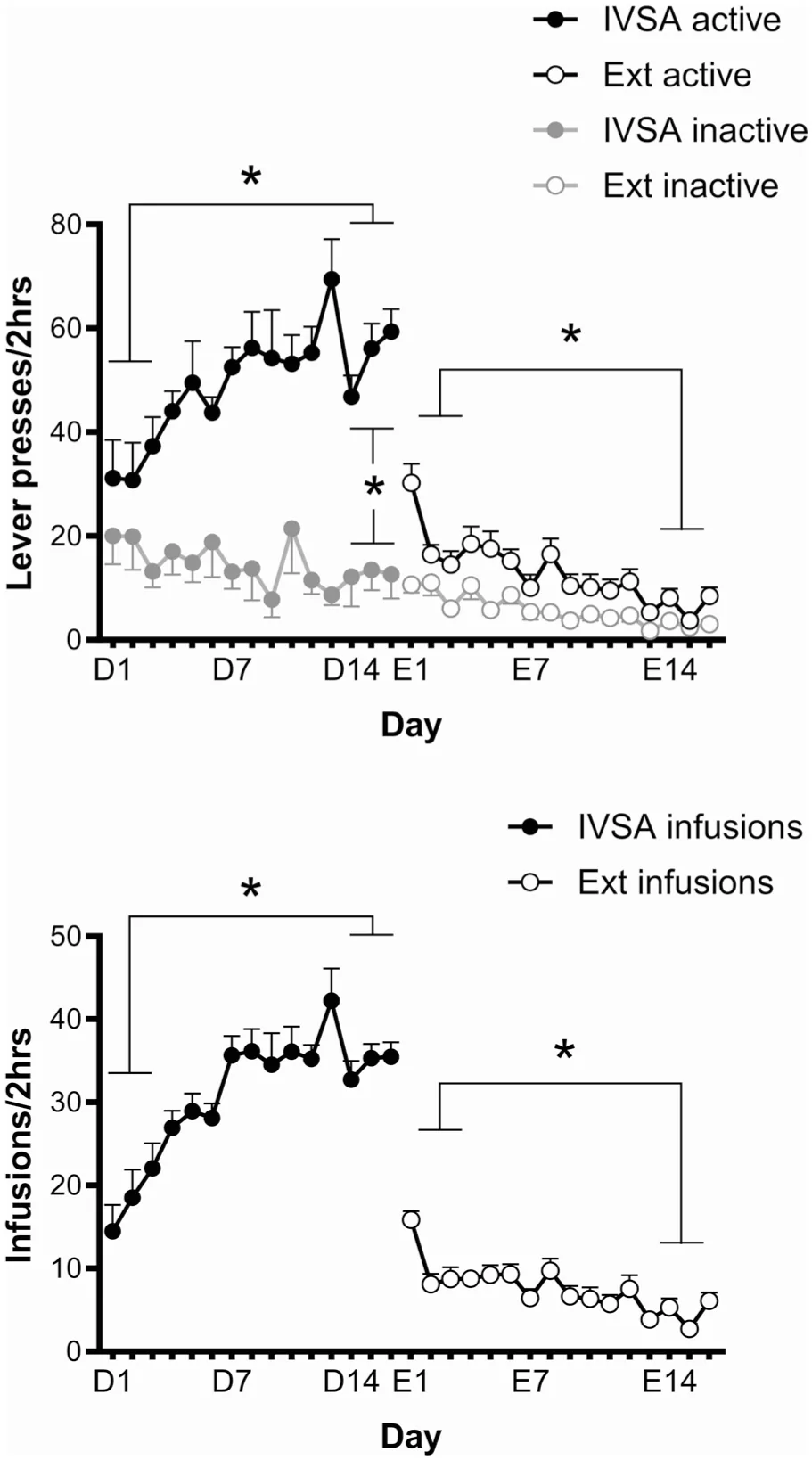
Number of active and inactive lever presses (top panel) and number of infusions (bottom panel) during 2-hour METH intravenous self-administration (IVSA, Day 1–15) and extinction (Ext, E1–E15) for the combined cohorts (*n* = 19) in Experiment 2. Data are mean ± SEM of *n* = 19 rats. *p < 0.05 for difference between the first 3 days and the last 3 days of IVSA or extinction or between the number of active lever presses and inactive lever presses.

The number of active lever presses (*F*(1, 18) = 9.87, *p* = 0.006, η_*p*_^2^ = 0.354) and the number of infusions (*F*(1, 18) = 27.62, *p* < 0.001, η_*p*_^2^ = 0.605) significantly increased from the first 3 days to the last three testing days of acquisition. In contrast, there was no difference in the number of inactive lever presses between the first and the last 3 days of acquisition (Figure 4, Cohort data Supplemental Figure 2). During the last 3 days of acquisition, the number of active lever presses was significantly greater than the number of inactive lever presses (*F*(1, 18) = 48.26, *p* < 0.001, η_*p*_^2^ = 0.728), showing that the rats had developed a significant preference for the lever associated with METH infusion (Figure 4, Cohort data Supplemental Figure 2).

During the extinction phase, there was a significant decrease in the number of active lever presses (*F*(1, 18) = 78.91, *p* < 0.001, η_*p*_^2^ = 0.814) and the number of infusions (*F*(1, 18) = 66.97, *p* < 0.001, η_*p*_^2^ = 0.788) between the first 3 days and the last 3 days of extinction testing. During the last 3 days of extinction testing, there was no difference between the number of active lever presses and inactive lever presses (Figure 4, Cohort data Supplemental Figure 2). This indicated that the rats had adequately extinguished their METH IVSA to begin reinstatement testing.

### Effect of i.c. THIP on METH-induced reinstatement

**Figure 5.**
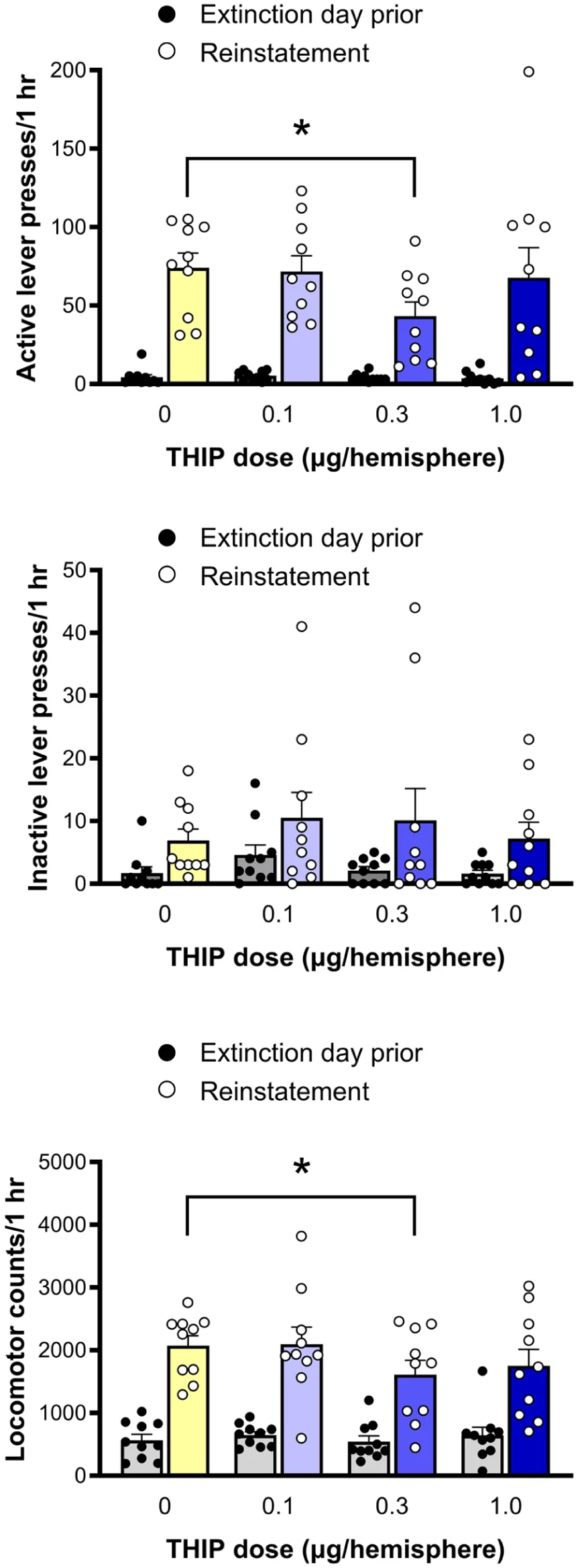
Number of active lever presses (top panel), inactive lever presses (middle panel) and locomotor counts (bottom panel) during 1 hour following i.c. microinjections of vehicle or THIP into the PLC during METH (1 mg/kg, i.p.) primed reinstatement sessions (*n* = 10). A 0.3 µg/hemisphere dose of THIP significantly reduced the number of active lever presses and locomotor activity when compared to vehicle (*p < 0.05). Data are mean ± SEM of *n* = 10 rats.

A METH priming injection significantly reinstated active lever pressing activity in rats when compared to the last extinction day prior (*F*(1, 9) = 51.7, *p* < 0.001, η_*p*_^2^ = 0.852; Figure 5). During the METH-primed reinstatement session rats responded significantly more on the active lever as opposed to the inactive (data not shown; *F*(1, 9) = 57.3, *p* < 0.001, η_*p*_^2^ = 0.864). While there was no significant effect of THIP dose on active responding in the omnibus ANOVA, because contrasts were planned a priori, planned pairwise comparisons were conducted. This showed that microinjection of THIP into the PLC at a dosage of 0.3 µg/per hemisphere significantly reduced METH-primed reinstatement. Specifically, while the number of active lever presses on the reinstatement day was significantly higher than that on the extinction day prior (*F*(1, 9) = 19.11, *p* = 0.002, η_*p*_^2^ = 0.680), this increase of active lever presses was significantly smaller than was observed following vehicle treatment (*F*(1, 9) = 8.31, *p* = 0.018, η_*p*_^2^ = 0.480). In contrast, THIP at a dose of 0.1 µg/per hemisphere and 1 µg/per hemisphere had no such effect (Figure 5). There were no effects of THIP on the number of inactive lever presses (Figure 5).

Active lever responding on the last METH-alone reinstatement session was significantly higher than on the extinction day prior (99.0 ± 17.4 vs 5.9 ± 1.1; *F*(1, 9) = 28.4, *p* < 0.001, η_*p*_^2^ = 0.760) indicating that repeated intracranial microinjections did not confound the ability of a METH prime to reinstate METH-seeking behaviour.

Comparison of locomotor activity on the reinstatement day compared to the last washout day revealed a significant increase (*F*(1, 9) = 56.0, *p* < 0.001, η_*p*_^2^ = 0.861) but no overall effect of THIP dose. However, planned comparison showed significantly reduced locomotor hyperactivity following the 0.3 µg dose of THIP (*F*(1 ,9) = 9.36, *p* = 0.014, η_*p*_^2^ = 0.510) but there was no effect of the 0.1 or 1.0 µg doses compared to vehicle (Figure 5).

### Effect of intracerebral THIP on cue-induced reinstatement

**Figure 6.**
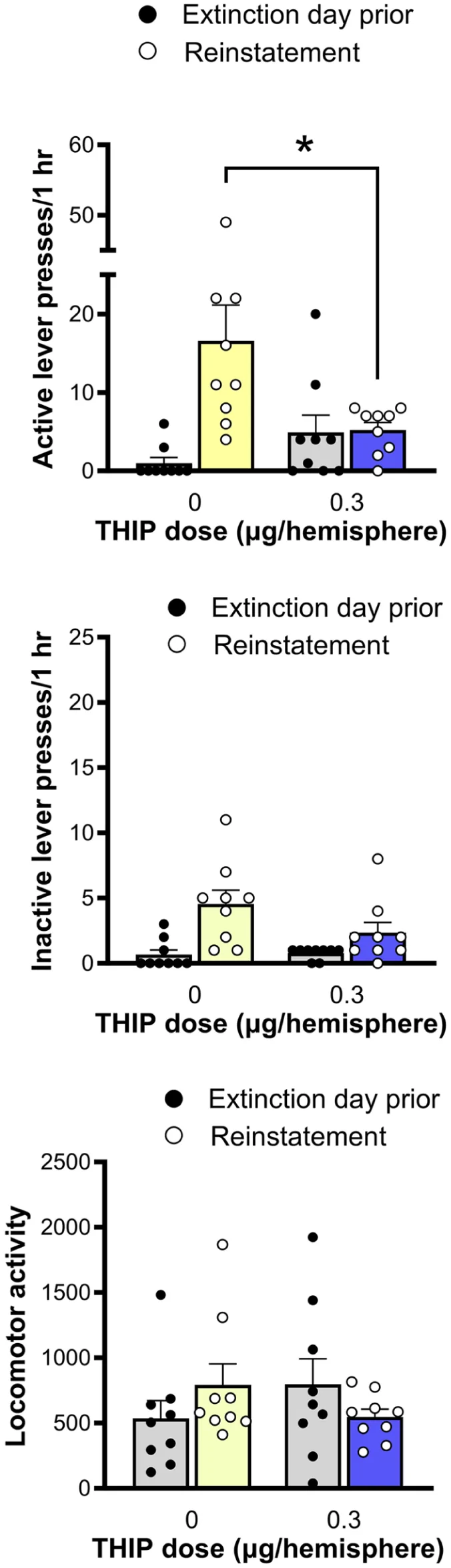
Number of active lever presses (top panel), inactive lever presses (middle panel) and locomotor counts (bottom panel) following i.c. microinjection of vehicle and 0.3 µg/hemisphere of THIP into the PLC during METH cue-induced reinstatement sessions (*n* = 9). THIP significantly reduced active lever pressing when compared to vehicle (*p < 0.05). Data are mean ± SEM of *n* = 9 rats.

Presentation of METH-associated cues reinstated active (METH-paired) lever pressing, as indexed by a significant increase in active lever responding on the vehicle reinstatement day compared to the extinction day prior (*F*(1, 8) = 7.76, *p* = 0.024, η_*p*_^2^ = 0.493; Figure 6). Additionally, a significant difference between active and inactive responding on the METH-primed vehicle reinstatement session was observed (*F*(1, 8) = 8.52, *p* = 0.019, η_*p*_^2^ = 0.516), signifying that METH-primed reinstatement was specific to the active lever (data not shown). Importantly, analysis of the change of the number of active lever presses between reinstatement sessions yielded a Day × Treatment interaction (*F*(1, 8) = 9.98, *p* = 0.013, η_*p*_^2^ = 0.555), with microinjection of THIP into the PLC at a dosage of 0.3 µg/per hemisphere reducing the increase in responding seen between the last day of washout and the cue-induced reinstatement session (Figure 6). During the reinstatement session, active lever pressing was significantly lower in rats injected with THIP compared to a vehicle dose (*F*(1, 8) = 6.99, *p* = 0.030, η_*p*_^2^ = 0.466; Figure 6). No significant difference between the number of inactive lever presses or locomotor activity counts on vehicle and THIP reinstatement days was observed (Figure 6).

Active lever responding on the last THIP-alone reinstatement session was not significantly different to the extinction day prior (3.6 ± 0.9 vs 4.9 ± 1.1) confirming that THIP alone did not reinstate any METH-seeking behaviour.

## Discussion

The present study investigated the effect of extrasynaptic GABA_A_ receptor modulation in the PLC of rats on METH-induced behavioural sensitisation and on relapse to METH-seeking behaviour elicited by a priming injection of the drug or by distinct METH-associated cues. The main findings were that, following the development of METH-induced sensitisation, THIP reduced METH-induced locomotor hyperactivity in a dose-dependent manner in the METH-METH group, where the highest dose of THIP produced the largest reduction in activity. Treatment with THIP did not reduce locomotor activity induced by acute METH in rats that were not sensitized. These findings demonstrate that extrasynaptic GABA_A_ activation in the PLC modulated METH-induced behavioural sensitisation. Planned pairwise comparisons revealed a selective reduction of METH-seeking behaviour at the 0.3 µg/hemisphere dose. Neither the lower (0.1 µg/hemisphere) nor higher (1.0 µg/hemisphere) doses significantly altered reinstatement, suggesting a non-linear, possibly *U*-shaped dose-response pattern. The 0.3 µg/hemisphere dose also significantly attenuated cue-induced reinstatement of METH-seeking behaviour.

These findings reflect previous research demonstrating that THIP administration can attenuate AMPH-induced behavioural sensitisation when administered into the mouse forebrain (Karler et al., 1997), as well as reduce cocaine and AMPH conditioned place preference when injected systemically, or into the NAc (Maguire et al., 2014). However, Siivonen and colleagues (2018) show that conditioned place preference to METH was not altered in δ-subunit GABA_A_R knock-out mice when compared to wild-type mice. Together these data may suggest that exogenously enhancing GABA tone at the δ-subunit GABA_A_Rs may effectively reduce METH behaviours, but that the absence of the receptor prior to METH exposure does not impact on the expression of METH reward. In line with this, the repeated injection of METH in δ-subunit GABA_A_R knock out mice displayed a greater sensitized locomotor effect than the wild-type mice, suggesting that the presence of the receptors may reduce the expression of METH sensitisation, although this was not statistically analysed by the group (Siivonen et al., 2018). A role of extrasynaptic δ-subunit GABA_A_Rs in psychosis was furthermore supported by brain post-mortem studies showing reduced GABAergic inhibition in the ACC in schizophrenia (Hashimoto et al., 2008; Maldonado-Aviles et al., 2009). The present results extend these previous findings and specifically suggest a role for δ-subunit GABA_A_Rs in the PLC in behavioural sensitization, a phenomenon associated with vulnerability to METH-induced psychosis, as well as in the reinstatement of METH-seeking behaviour, modelling relapse in METH use disorder. Indeed, there is recent evidence that polymorphisms of the δ-subunit of GABA_A_R may be implicated in METH dependence or psychoses, in a sex-dependent manner (Xie et al., 2023).

### Different THIP dose-response relationship between Experiment 1 and Experiment 2

The results of Experiment 1 showed that intra-PLC THIP administration dose-dependently modulated METH-induced locomotor hyperactivity but only when animals had been treated according to the METH sensitisation paradigm. These findings are consistent with previous research (Wearne et al., 2015) suggesting that alterations to the GABAergic system within the prefrontal network result from chronic METH administration, and that such alterations may only manifest in behavioural changes when excessive excitatory input occurs, such as that from a METH challenge (Jodo et al., 2003; Steketee, 2003). The lack of effect of THIP on locomotor activity of rats treated with METH only on challenge day (Sal/METH group) may be explained by a lack of plasticity and/or toxic alterations in the PLC or alterations to the GABAergic system caused by chronic METH treatment (Jodo et al., 2003; Pierce and Kalivas, 1997; Steketee, 2003; Wearne and Cornish, 2019).

The results of Experiment 2 demonstrate that specific activation of PLC δ-subunit GABA_A_Rs attenuated relapse to METH-seeking elicited by a METH prime or associated cues. These findings are in line with previous research showing that elevating non-specific GABA_A_R activation, to inhibit the PLC, reduced METH-primed and METH-cued relapse to drug-seeking behaviour (Rocha and Kalivas, 2010), however the current study specifically targeted δ-subunit GABA_A_Rs to inhibit METH-seeking behaviour. This suggests that tonic GABA activity in the PLC may impact on other circuits involved in drug reward, such as the NAc or striatum, to reduce psychostimulant-like effects and addictive behaviours (Luscher et al., 2020).

A significant reduction in METH-induced active lever pressing was observed following the infusion of a medium (0.3 µg/hemisphere) but not a higher (1.0 µg/hemisphere) dose of THIP when compared to vehicle control. In isolation, these results pertaining to METH reinstatement might be explained by partial agonist properties of THIP at GABA_A_R isoforms other than the δ-subunit, such as synaptic GABA_A_R containing γ-subunits, at higher doses interfering with a more selective effect at 0.3 µg/hemisphere (Meera et al., 2011; Mortensen et al., 2010). However, based on the results from Experiment 1, a dose-dependent linear attenuation of METH-primed reinstatement (active responding for the drug) could be expected following the three increasing doses of THIP into the PLC. This dose-response discrepancy suggests differences in neuronal circuitry and, by extension, the role of δ-subunit GABA_A_Rs, between models of METH psychosis and METH-induced reinstatement of drug seeking behaviour. In addition, in Experiment 2, the IVSA paradigm was used to directly assess addictive drug-seeking behaviour, where rats were required to learn to actively self-administer infusions of METH by lever pressing over an extended period of time (approximately 3 weeks). In contrast, the rats in Experiment 1 and a previous study (Karler et al., 1997) underwent passive, experimenter delivered, administration of METH over approximately 1 week to induce a progressively enhanced behavioural response to the drug after prolonged abstinence (Jaehne et al., 2023; Pierce and Kalivas, 1997; Wearne et al., 2015). IVSA rats in Experiment 2 likely experienced a higher degree of METH exposure compared to rats in Experiment 1 that underwent the behavioural sensitization protocol. Specifically, cumulative exposure to METH experienced by rats in Experiment 1 would be 27 mg/kg i.p. over 1 week, whereas the rats in Experiment 2 would be exposed to around 53 mg/kg i.v., based on approximately 35 infusions per day for 15 days (see Figure 4) at 0.1 mg/kg/infusion. In addition, i.p. and i.v. administration are known to lead to different drug pharmacokinetics and pharmacodynamics (for references, see (Al Shoyaib et al., 2019)) and different neuroadaptive alterations are known to occur from passive compared to active models of drug self-administration (Knackstedt and Kalivas, 2007; Stefanski et al., 1999). An additional difference between the models was extinction training in the IVSA rats when compared to withdrawal in the sensitised group. While either may induce changes to GABAergic signaling in the PLC, experience dependent receptor changes associated with active learning are likely different to those induced by drug withdrawal (Roberts-Wolfe et al., 2018). These differences in methodology could explain why the pharmacological actions of THIP interacted differently between these two independent, yet related, models of METH-induced behaviour.

### Potential cellular mechanisms involved in the effect of THIP

There are a number of potential explanations for the present findings, that THIP administration into the PLC attenuated both hyperlocomotor responding following METH sensitisation, as well as METH-prime and cued reinstatement of drug seeking behaviour. The most likely mechanism can be understood through the functionality of extrasynaptic GABA_A_Rs. These specific receptors are persistently activated by the binding of ambient GABA concentrations that induce a slow and constant ‘tonic’ inhibitory input into the cell (Farrant and Nusser, 2005). These ambient GABA concentrations located in extracellular space are derived from the ‘spill over’ of excess GABA in the synaptic cleft (Belelli et al., 2009; Whissell et al., 2015). Thus, elevated quantities of GABA are necessary to ‘spill over’ and activate extrasynaptic GABA_A_Rs. However, previous studies have observed reductions in GABA synthesis (Wearne et al., 2016) and GABA transmission (Bu et al., 2013) within the PLC following chronic METH exposure. These findings imply that extended exposure to METH may cause a reduction in GABA signalling and subsequent ‘spill over’ to activate extrasynaptic GABA_A_Rs to enable behavioural control, primarily through inhibition of glutamatergic pyramidal cells from the PLC to NAc. It is plausible to suggest that these receptors are not adequately activated in the PLC, facilitating METH sensitisation and relapse to METH-seeking. Therefore, elevating extrasynaptic GABA_A_R activity may serve to restore or ‘normalise’ PLC activity through enhanced tonic GABAergic inhibition, thereby suppressing METH-induced hyperlocomotion or METH-seeking behaviours upon exposure to a METH-prime or METH-paired cue.

### Functional pathways involved in the effect of THIP following METH exposure

The PLC has dense glutamatergic efferent projections to the NAc, specifically the inner sub territory of this region known as the NAc core (Hoover and Vertes, 2007). Specifically with respect to METH-primed reinstatement, it is important to note that this PLC-NAc glutamate projection is directly implicated in drug-primed reinstatement (Kalivas and McFarland, 2003; McFarland et al., 2003; Schmidt et al., 2005). For example, upon administration of a cocaine prime, amplified PLC activation acts to promote cocaine seeking by stimulating the glutamatergic projection from the PLC to the NAc core (McFarland et al., 2003; Park et al., 2002). This increases glutamate release into the accumbens region, which has been shown to initiate renewed drug-seeking produced by systematic cocaine administration (Cornish and Kalivas, 2000). Indeed, cocaine-primed reinstatement was accompanied by augmented glutamate release in the NAc core, an effect that was reduced via pharmacological inhibition of the mPFC, in which the PLC is located (McFarland et al., 2003). In light of the current findings for METH-primed reinstatement, it is conceivable that activation of this excitatory PLC-NAc glutamatergic projection becomes reduced following the restoration of inhibitory GABA tone produced by THIP infusion into the PLC. By pharmacologically elevating tonic GABA inhibition into the PLC this may have attenuated or ‘silenced’ the activity of glutamate efferents to the NAc core and in turn, fail to increase excitatory glutamate transmission into this region to promote drug-seeking behaviour. As a result, there would be an observed reduction in relapse propensity following a METH prime, which may explain the present METH-primed reinstatement findings. However, an attenuation in cue-induced reinstatement cannot be solely explained based on the functional connection between the PLC and NAc core, as pharmacological inactivation of the NAc core does not block reinstatement to drug-paired reinforcers (Grimm and See, 2000). Compared to drug-primed relapse, cue-induced reinstatement is based on a process of associative learning where a previously neutral stimuli paired with repeated drug consumption, acquires incentive-motivational properties to induce drug craving and elicit relapse to previous drug-seeking behaviour (Campus et al., 2019; See, 2005). Thus, cued-relapse encompasses a brain circuit involved in associative learning, which predominantly embodies the PFC, amygdala, NAc and paraventricular nucleus of the thalamus (Campus et al., 2019; See, 2005). In relation to the current findings, it is plausible that an elevation in tonic GABA current within the PLC acts to ‘silence’ or reduce the stimulation of this circuit.

### Limitations

A limitation of this study is that we only used male rats. Several studies have shown sex differences in behavioural and neurochemical effects of prolonged METH exposure (Cornish and Prasad, 2021; Dluzen and Liu, 2008; Hogarth et al., 2022; Jaehne et al., 2023; Ruda-Kucerova et al., 2015). A preliminary study with human subjects also suggests that different polymorphisms of the delta subunit of the GABA receptor may be associated with METH dependence or METH psychosis in females and males, respectively (Xie et al., 2023). Therefore, further studies are needed to investigate the effect of THIP in female as well as male rats.

Given the heterogeneous structure and diversity of cell types within the PFC (Lewis et al., 2005), it is furthermore important to understand where THIP may be altering cortical excitability. Some evidence suggests that GABA produces tonic activity primarily around the body of the cell (soma), as opposed to the dendritic spines extending from the neuron (Soltesz et al., 1995). Pyramidal cells are the primary cells within the PFC, and are critical to generating neuronal activity for appropriate behavioural output (Lewis et al., 2005). It is reasonable to postulate that ‘basket’ type cells, which contain GABA and project onto the soma of pyramidal cells in the mPFC (Yang et al., 2013), may generate tonic inhibition onto pyramidal cells. In line with this theory, high doses of METH reduced the number of synaptic connections onto the soma of pyramidal cells in the mPFC (Brummelte et al., 2007), suggesting extrasynaptic GABA_A_R impairment could occur around the body of pyramidal cells. However, this hypothesis has not yet been directly tested.

An additional limitation of this study was the difference in strength of reinstatement for a METH-prime and METH-cue. In terms of the number of active lever presses, reinstating was markedly weaker for cue-induced reinstatement than for METH-primed reinstatement. The explanations for this finding are twofold; (1) Drug-primed reinstatement involves the immediate euphoric ‘feeling’ of the drug, which is known to robustly reinstate active pursuit of drug-seeking behaviour (Schmidt et al., 2005); (2) The cues employed in this study to trigger cued-relapse were lacking in strength. These cues included a contingent light stimulus that became illuminated upon active lever depression and a non-contingent peppermint oil odour that symbolised the METH-related context. Considering only one cue was contingent with an active response, yet the smell signified drug context, applying another contingent cue would be beneficial to strengthen cued-reinstatement.

## Conclusions

The present study demonstrated that activation of extrasynaptic GABA_A_ receptors using the GABA_A_ δ-subunit receptor agonist THIP, attenuated the sensitised response to a METH challenge, suggesting that prefrontal tonic GABA_A_ activity may be involved in the underlying pathology of vulnerability to METH-induced psychosis. The present investigation furthermore revealed that extrasynaptic GABA_A_R activation within the PLC alleviates relapse propensity following exposure to METH cues or re-exposure to the drug. Thus, through THIP administration, modulation of δ-subunit GABA_A_Rs in the PLC, a critical node in the effects of prolonged METH exposure, may offer promising utility in the development of efficacious pharmacotherapies aiming to alleviate effects of prolonged METH use.

## Supplemental Material

sj-docx-2-jop-10.1177_02698811261430509 – Supplemental material for Administration of an extrasynaptic δ-subunit GABAA receptor agonist in the prelimbic cortex attenuates methamphetamine sensitisation and relapse to methamphetamine-seeking behaviour in male rats Supplemental material, sj-docx-2-jop-10.1177_02698811261430509 for Administration of an extrasynaptic δ-subunit GABAA receptor agonist in the prelimbic cortex attenuates methamphetamine sensitisation and relapse to methamphetamine-seeking behaviour in male rats by Maarten van den Buuse, Jessica Roberts, Michael T. C. Hunter, Gracie L. Hay, Nicholas A. Everett, Sarah J. Baracz, Travis A. Wearne and Jennifer L. Cornish in Journal of Psychopharmacology

sj-jpg-1-jop-10.1177_02698811261430509 – Supplemental material for Administration of an extrasynaptic δ-subunit GABAA receptor agonist in the prelimbic cortex attenuates methamphetamine sensitisation and relapse to methamphetamine-seeking behaviour in male rats Supplemental material, sj-jpg-1-jop-10.1177_02698811261430509 for Administration of an extrasynaptic δ-subunit GABAA receptor agonist in the prelimbic cortex attenuates methamphetamine sensitisation and relapse to methamphetamine-seeking behaviour in male rats by Maarten van den Buuse, Jessica Roberts, Michael T. C. Hunter, Gracie L. Hay, Nicholas A. Everett, Sarah J. Baracz, Travis A. Wearne and Jennifer L. Cornish in Journal of Psychopharmacology
