# Supplementary material for: Administration of an extrasynaptic δ-subunit GABAA receptor agonist in the prelimbic cortex attenuates methamphetamine sensitisation and relapse to methamphetamine-seeking behaviour in male rats
Source: J Psychopharmacol. 2026 Mar 28;40(9):1480–92. doi: 10.1177/02698811261430509 (PMC13490680; doi:10.1177/02698811261430509)
Supplement: Supplementary file 1 [file sj-docx-2-jop-10.1177_02698811261430509.docx]

- Supplementary data -

Bregma +4.68mm

Bregma +4.20mm

Bregma +3.72mm

Bregma +3.24mm

Bregma +3.00mm

Bregma +2.56mm

Supplementary Figure 1

Location of microinjection cannula placements for Experiment 1 (left panel) and Experiment 2 (right panel). Rats with either bilateral or unilateral misplaced intracranial cannulas, located outside the PLC, were omitted from data analysis.
